# mTOR pathway: A key player in diabetic nephropathy progression and therapeutic targets

**DOI:** 10.1016/j.gendis.2024.101260

**Published:** 2024-03-08

**Authors:** Jingxuan Shi, Xinze Liu, Yuanyuan Jiao, Jingwei Tian, Jiaqi An, Guming Zou, Li Zhuo

**Affiliations:** a Department of Nephrology, China-Japan Friendship Hospital, Beijing 100029, China; b China-Japan Friendship Institute of Clinical Medical Sciences, Beijing 100029, China; c Beijing University of Chinese Medicine China-Japan Friendship Clinical Medical College, Beijing 100029, China; d Department of Nephrology, Fuwai Hospital, Chinese Academy of Medical Science, Beijing 100037, China; e Department of Nephrology, Beijing Sixth Hospital, Beijing 100007, China; f Capital Medical University China-Japan Friendship School of Clinical Medicine, Beijing 100029, China; g China-Japan Friendship Clinic Medical College, Peking University, Beijing 100191, China

**Keywords:** Bibliometrics, Diabetic nephropathy, Glomerular endothelial cell, Immune cell, Mesangial cell, mTOR, Podocyte, Renal tubular epithelial cell

## Abstract

Diabetic nephropathy is a prevalent complication of diabetes and stands as the primary contributor to end-stage renal disease. The global prevalence of diabetic nephropathy is on the rise, however, due to its intricate pathogenesis, there is currently an absence of efficacious treatments to enhance renal prognosis in affected patients. The mammalian target of rapamycin (mTOR), a serine/threonine protease, assumes a pivotal role in cellular division, survival, apoptosis delay, and angiogenesis. It is implicated in diverse signaling pathways and has been observed to partake in the progression of diabetic nephropathy by inhibiting autophagy, promoting inflammation, and increasing oxidative stress. In this academic review, we have consolidated the understanding of the pathological mechanisms associated with four distinct resident renal cell types (podocytes, glomerular mesangial cells, renal tubular epithelial cells, and glomerular endothelial cells), as well as macrophages and T lymphocytes, within a diabetic environment. Additionally, we highlight the research progress in the treatment of diabetic nephropathy with drugs and various molecules interfering with the mTOR signaling pathway, providing a theoretical reference for the treatment and prevention of diabetic nephropathy.

## Introduction

Diabetic nephropathy (DN) is recognized as the primary microvascular complication of diabetes mellitus and is the leading cause of chronic kidney disease and end-stage renal disease.^1^^,^^2^ The clinical manifestations of DN encompass progressive proteinuria, reduced glomerular filtration rate,^3^ glomerular hypertrophy, thickening of the glomerular basement membrane, mesangial hyperplasia, and podocyte loss.^4^, ^5^, ^6^ Currently, the management of DN primarily involves nutritional interventions and control of blood glucose and hypertension, including the administration of hypoglycemic agents that directly impact renal function and drugs that inhibit the renin-angiotensin system.^7^^,^^8^ However, the current treatments for DN are limited to delaying the onset and progression of the disease and do not effectively reverse renal injury and dysfunction or prevent the development of end-stage renal disease.^9^^,^^10^ The pathogenesis of DN is complex and involves various factors such as oxidative stress, inflammatory cascade, and metabolic pathway disorders caused by persistent hyperglycemia.^11^^,^^12^ Therefore, there is a need to investigate the underlying mechanisms of DN to develop novel approaches for the prevention and treatment of this condition. Currently, the precise molecular pathways involved in the pathogenesis of DN remain to be elucidated. There has been significant research conducted on the precise molecular pathways implicated in the development of DN. Presently, a growing body of evidence indicates a robust correlation between mammalian target of rapamycin (mTOR) complexes and the presentation of nephrotic symptoms in diverse forms of DN.^13^^,^^14^ Pharmacological agents, such as rapamycin, that disrupt the mTOR signaling pathway have exhibited encouraging therapeutic outcomes in preclinical experimental models of DN.^15^ Consequently, directing interventions toward the mTOR pathway presents considerable promise for the management of DN.

The presence of the mTOR has been observed in yeast, where it functions as a binding protein for rapamycin across various species, including mammals.^16^^,^^17^ mTOR belongs to the phosphatidylinositol-associated protein kinase family and acts as a serine/threonine protein kinase. It is comprised of two distinct protein complexes, namely mTOR complex 1 and mTOR complex 2.^18^ Each complex carries out specific functions by phosphorylating different substrates. Notably, growth factors such as insulin, amino acids, and sugars enhance the activity of mTOR complex 1. mTOR complex 1 phosphorylates its substrate, thereby facilitating cell differentiation and growth, augmenting intracellular anabolism, and impeding catabolism, including autophagy.^19^^,^^20^ mTOR complex 2, on the other hand, primarily serves as a substrate for phosphorylation and activation of other kinases, promoting cytoskeleton reorganization and cell proliferation, while inhibiting cell death.^19^ Previous research has demonstrated that hyperglycemia, obesity, hyperinsulinemia, and excessive amino acids all contribute to increased mTOR activity in the kidney.^21^, ^22^, ^23^ Excessive activation of mTOR can disrupt the balance of protein synthesis, leading to metabolic disorders and increased endoplasmic reticulum stress and oxidative stress within the cells.^24^, ^25^, ^26^

In this academic review, we consolidate the understanding of the pathological mechanisms associated with four distinct resident renal cell types (podocytes, glomerular mesangial cells, renal tubular epithelial cells, and glomerular endothelial cells), as well as macrophages and T lymphocytes, within a diabetic environment. Additionally, we summarize the current research progress in the treatment of DN using drugs and various interventions targeting the mTOR signaling pathway.

## Bibliometric analysis of research on mTOR in diabetic nephropathy

### A brief introduction and purpose of bibliometric analysis

In recent years, numerous articles have been published by researchers both within and outside the country, delving into the mTOR pathway in the context of DN progression and therapeutic targets. To enhance the systematic and comprehensive nature of this review, we conducted a bibliometric analysis to comb through and summarize the existing studies.

## Methods

We performed in the Web of Science up to 1st August 2023 using keywords “diabetic kidney disease”, “diabetic nephropathy”, “mTOR”, “autophagy”, and “rapamycin”. We included studies of the mTOR pathway in DN and excluded reduplicative publications, conference address, and coverages. Subsequently, we used VOSviewer 1.6.19 (Leiden University, Leiden, Netherlands) to extract the authors, countries, institutions, and keywords of included literature; then, network maps and cluster analysis were generated. Furthermore, we produced a density map illustrating the distribution of keywords. It is worth mentioning that in network maps, the nodes were regarded as representatives of the quantity or occurrence rate of analyzed objects, while the connections between nodes denoted various relationships such as co-occurrence. Similarly, in the density map, the intensity of color is directly correlated with the frequency of the keywords.

## Results

### Search results

There were 465 publications from core journals. We excluded 11 publications in accordance with the exclusion criteria and included 454 publications in the end.

### Keywords

**Figure 1:**
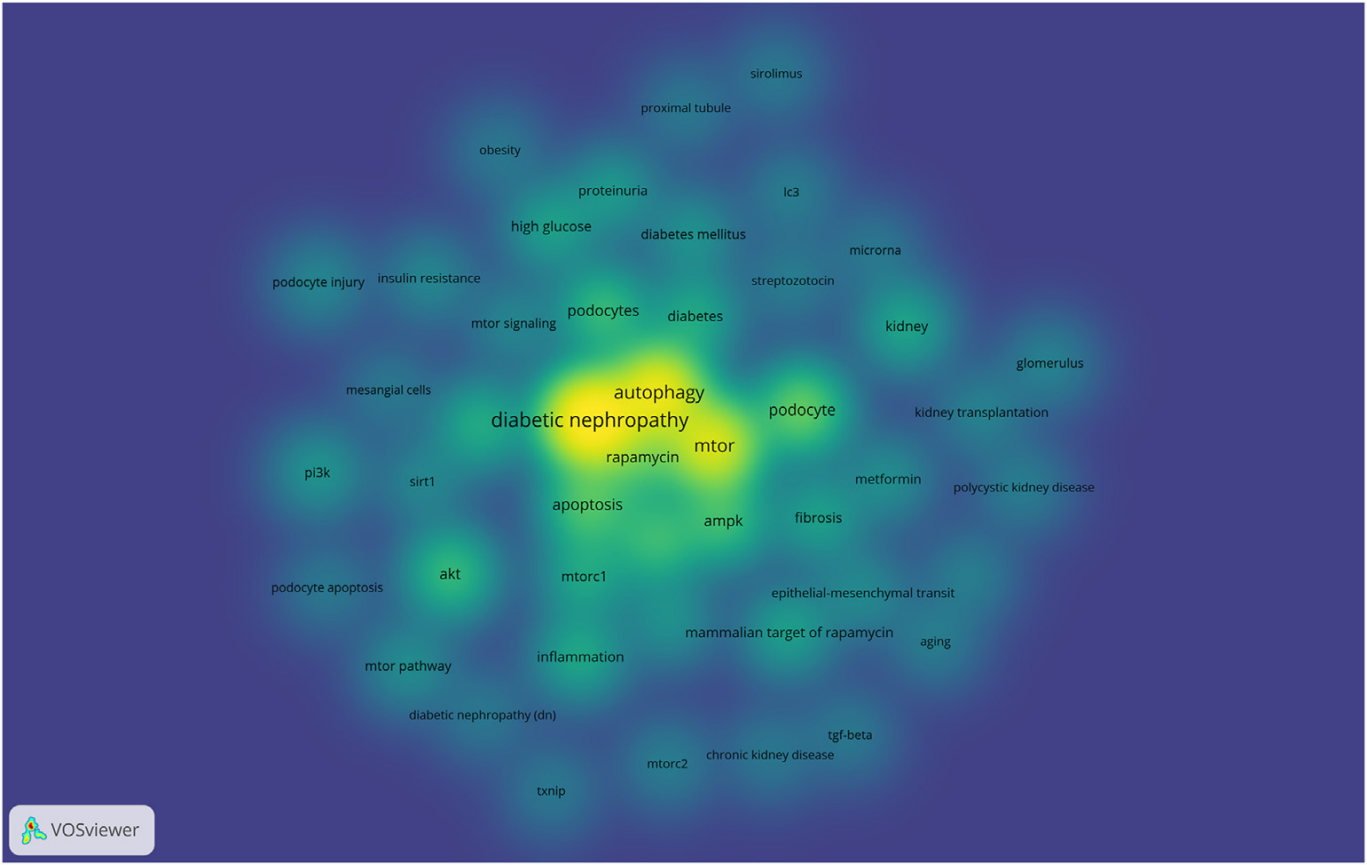
Density map of main keywords. The brightness of the color is positively correlated with the frequency of keywords.

Through the co-occurrence analysis of keywords, we could quickly capture research hotspots in a certain field. Table 1 shows the top 20 high-frequency keywords in research on the mTOR pathway in DN. Among the identified keywords, “diabetic nephropathy”, “autophagy”, and “mTOR” were found to have occurred more than 50 times, indicating their significance as the primary research focus in the field of mTOR in the context of DN. A density map was constructed to visually represent the distribution of keywords with an occurrence frequency of 5 or higher (Fig. 1). The density map consisted of a total of 47 nodes, with the most prominent ones being “diabetic nephropathy” and “autophagy”, positioned centrally and surrounded by related terms such as “podocyte”, “apoptosis”, “AMPK”, “AKT”, and “rapamycin”. Conversely, the periphery of the map exhibited a darker shade, encompassing terms such as “TXNIP”, “TGF-beta”, and “sirolimus”.

**Table 1:** Top 20 high-frequency keywords.

Rank	Keywords	Counts	Rank	Keywords	Counts
1	diabetic nephropathy	157	11	oxidative stress	21
2	autophagy	110	12	diabetes	19
3	mTOR	95	13	kidney	19
4	podocyte	43	14	mtorc1	18
5	apoptosis	35	15	inflammation	18
6	AMPK	29	16	fibrosis	16
7	AKT	28	17	high glucose	16
8	Diabetic kidney disease	28	18	mammalian target of rapamycin	16
9	podocytes	28	19	proteinuria	13
10	rapamycin	28	20	pi3k	12

### Authors

**Figure 2:**
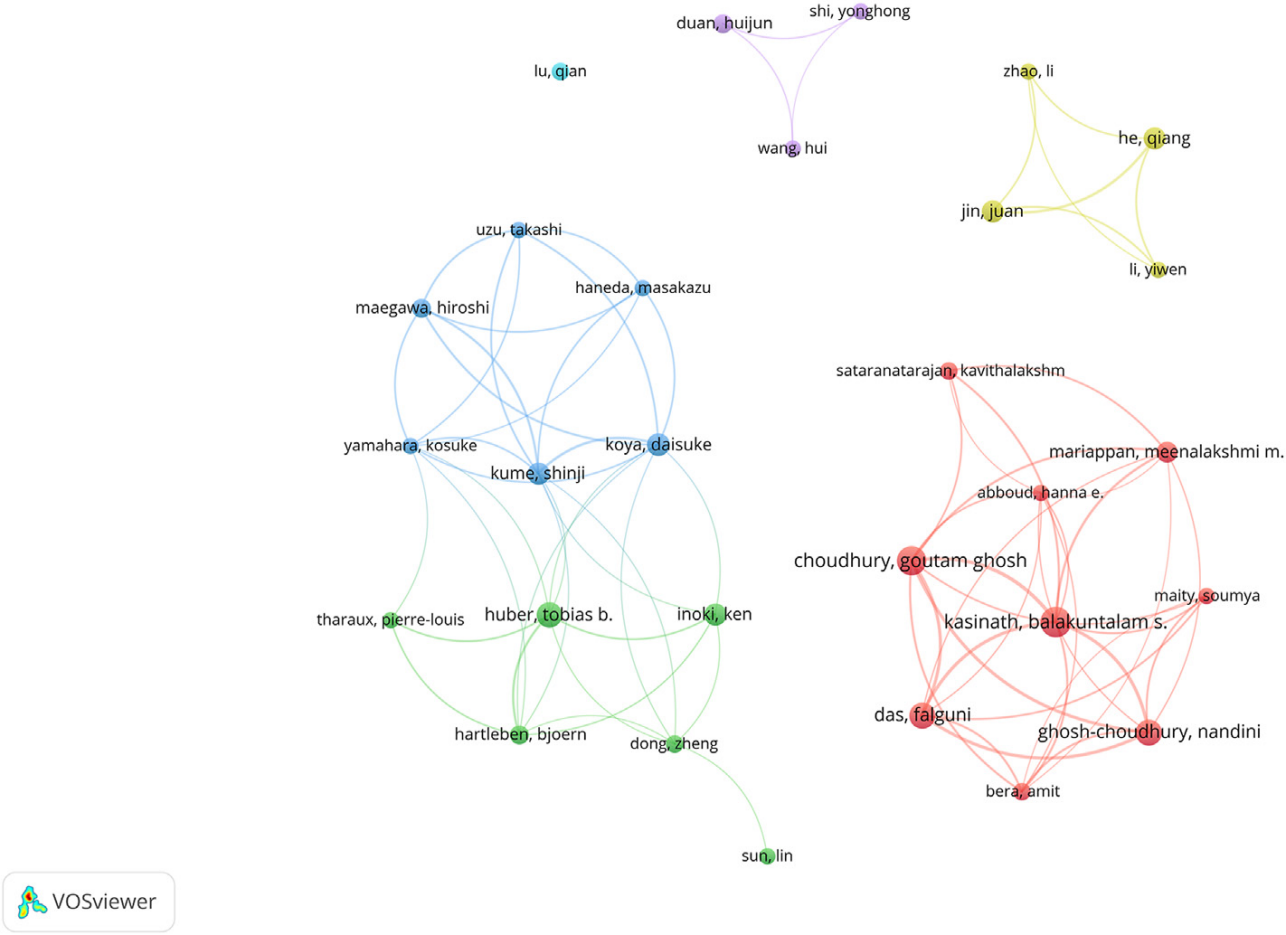
Network map of 29 authors with frequency greater than five. Nodes represent authors, and the larger the node, the higher the number of papers. The lines represent collaborations with other authors, and the thicker the line, the higher the cooperation frequency. Color represents clustering, and nodes with the same color belong to the same cluster.

A total of 2549 authors have been busy with relevant studies of the mTOR pathway in DN. We build a collaborative network based on authors whose number of published papers is more than or equal to 5 (Fig. 2). Balakuntalam S Kasinath, Goutam Ghosh Choudhury, and Falguni Das have the largest nodes because they publish the most related publications. Besides, we observed close collaboration among multiple authors. For example, Balakuntalam S Kasinath has close cooperation with Goutam Ghosh Choudhury and Hanna E Abboud; Tobias B Huber has active cooperation with Ken Inoki and Bjoern Hartleben.

## Countries and institutions

**Figure 3:**
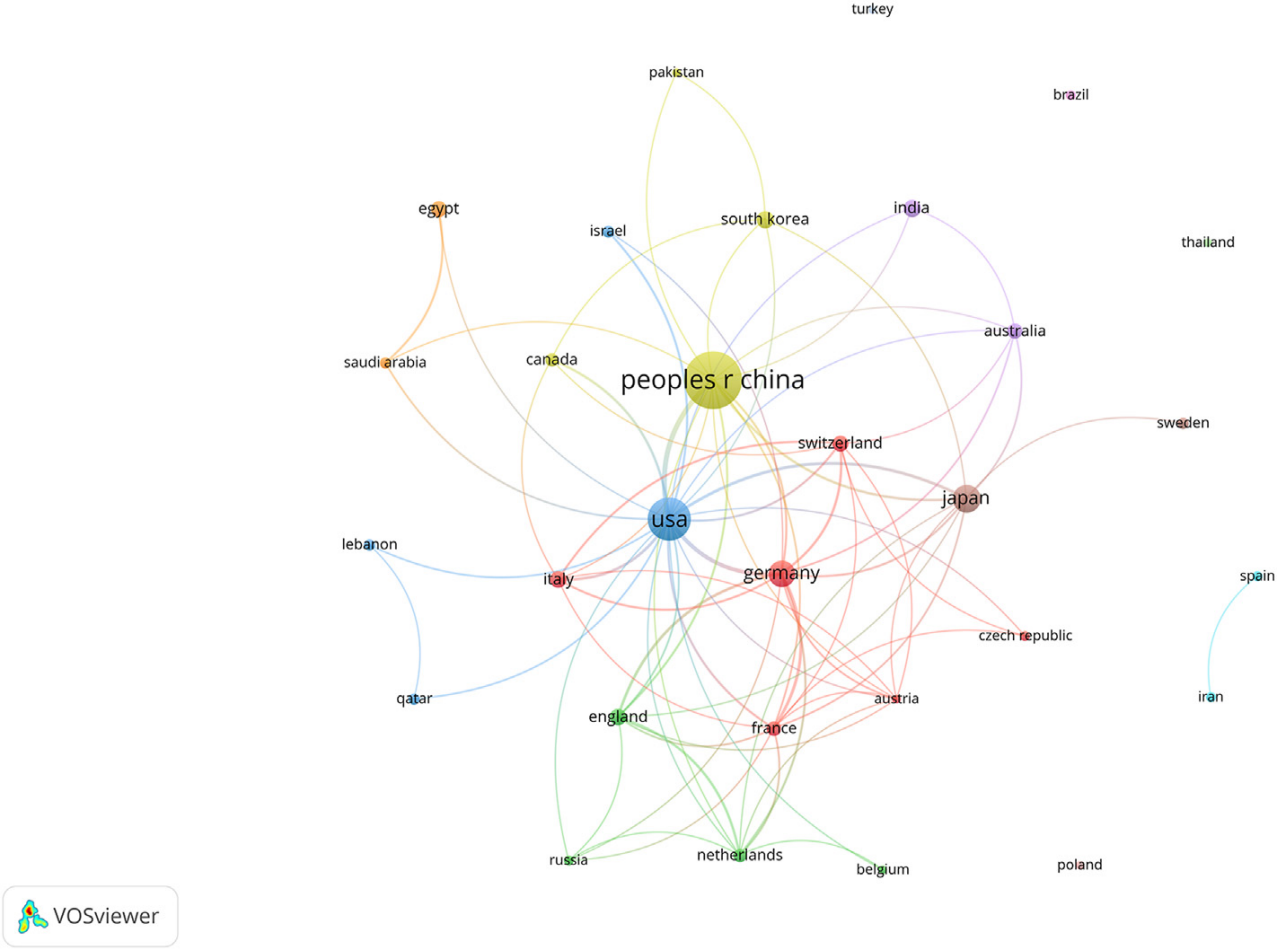
Network map of 31 countries with a frequency greater than two. Nodes represent countries, and the larger the node, the higher the number of papers. The lines represent collaborations with other countries, and the thicker the line, the higher the cooperation frequency. Color represents clustering, and nodes with the same color belong to the same cluster.

These publications came from 41 countries and 620 institutions. Among the countries, the country with the largest number of publications is China (*n* = 235, 51.8%), followed by The United States (*n* = 114, 25.1%), Japan (*n* = 38, 8.4%), and Germany (*n* = 33, 7.3%). Subsequently, we filtered and visualized 31 countries based on the number of publications more than or equal to 2, and constructed a collaborative network based on the number and relationship of publications in each country (Fig. 3). Notably, there is a lot of active cooperation between different countries. For example, China has close cooperation with The United States, Germany, and England.

**Figure 4:**
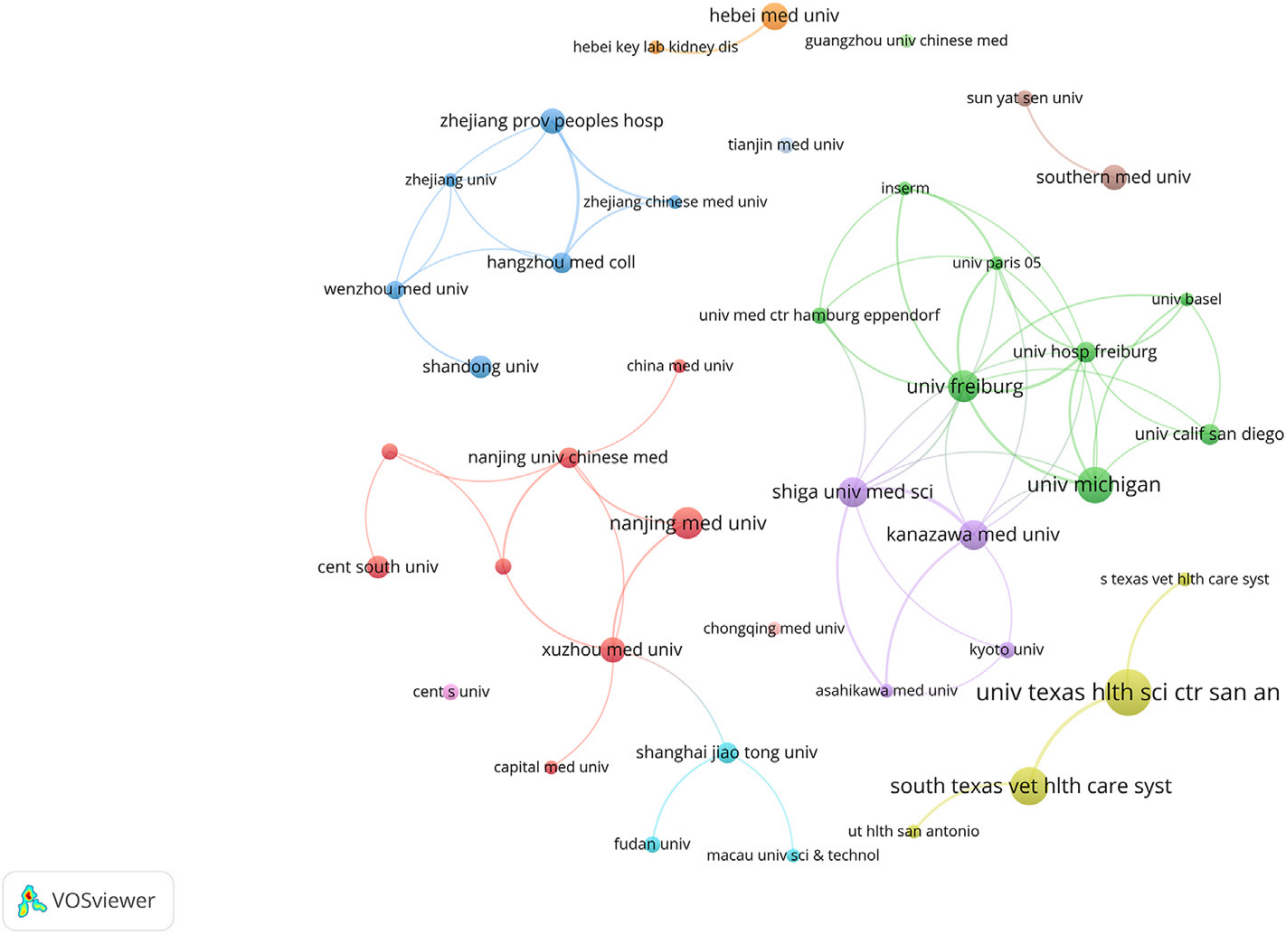
Network map of 41 institutions with a frequency greater than five. Nodes represent institutions, and the larger the node, the higher the number of papers. The lines represent collaborations with other institutions, and the thicker the line, the higher the cooperation frequency. Color represents clustering, and nodes with the same color belong to the same cluster.

The top 10 institutions are located in 4 countries. The three institutions that published the most relevant papers are The University of Texas Health Science Center at San Antonio (*n* = 20, 4.4%), South Texas Veterans Health Care System (*n* = 16, 3.5%), and The University of Michigan (*n* = 15, 3.3%). Subsequently, we selected 41 institutions based on the minimum number of publications equal to 5 for visualization, and constructed a collaborative network based on the number and relationship of publications of each institution (Fig. 4). The cooperation between The University of Michigan, University of Freiburg, Kanazawa Medical University, and Shiga University of Medical Science is very close. In addition, we note that Guangzhou University of Chinese Medicine and Tianjin Medical University has no partnership with other institutions.

## Discussion

The study examined the keywords, authors, institutions, and countries associated with researchers studying the mTOR pathway in DN using bibliometric analysis. This analysis provided an up-to-date overview of the research conducted on mTOR in DN globally. Our investigation of key terms revealed that “autophagy”, “podocyte”, “apoptosis”, “AMPK”, and “AKT” are fundamental keywords within this domain. Prior research has elucidated the significant correlation between autophagy and the advancement of DN, while also highlighting the crucial role played by the AMPK-AKT-mTOR pathway in autophagy regulation. In forthcoming studies, we anticipate broadening the scope of investigation pertaining to these fundamental keywords to offer enhanced understanding and methodologies for the pathogenesis and treatment of DN. Concurrently, by scrutinizing the authors, countries, and research institutions involved, we unveil experts and institutions possessing profound insights in this domain, thereby furnishing valuable guidance for future research endeavors and collaborative prospects. In general, we can focus on interdisciplinary cooperation to facilitate them.

## The mechanisms and therapeutic implications of mTOR signaling pathway in diabetic nephropathy

### Podocytes and mTOR

**Figure 5:**
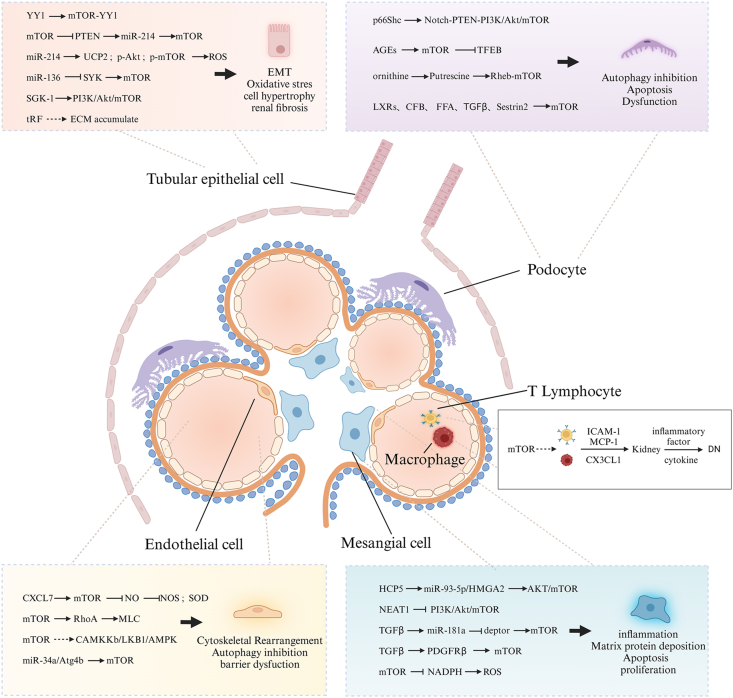
The pathological mechanisms of diabetic nephropathy associated with mTOR in distinct resident renal cells, macrophages, and T lymphocytes (created with BioRender.com). mTOR regulates different molecules and pathways or is regulated by them in different cells, resulting in different pathological changes in different cells and participating in the progression of diabetic nephropathy. AGEs, advanced glycation end products; CAMKKb, calcium/calmodulin-dependent protein kinase b; CFB, complement factor B; CX3CL1, fractalkine; CXCL7, platelet microparticle-derived chemokine ligand 7; FFC, free fatty acids; HCP5, HLA complex P5; ICAM-1, intercellular adhesion molecule-1; LKB1, liver kinase B1; LXR, liver X receptor; MCP-1, monocyte/macrophage chemoattractant protein-1; MLC, myosin light chain; NEAT1, nuclear enriched abundant transcript 1; NOS, nitric oxide synthase; p66Shc, reactive oxygen species regulatory protein; PDGFR-β, platelet-derived growth factor receptor β; PTEN, phosphatase and tensin homolog; Rheb, sensor GTPase Ras homologue; ROS, reactive oxygen species; SYK, spleen tyrosine kinase; SGK-1, serum and glucocorticoid-induced kinase 1; SOD, superoxide dismutase; TGF-β, transforming growth factor β; tRF, transfer RNA derived fragment; UCP2, uncoupling protein 2; YY1, Yin Yang 1.

Podocytes, highly specialized epithelial cells with limited proliferative capacity, are closely adhered to the glomerular basement membrane^27^ and play a crucial role in maintaining the integrity of the glomerular filtration barrier.^28^^,^^29^ Damage and apoptosis of podocytes can disrupt the glomerular filtration barrier,^30^ resulting in proteinuria, renal lesions, and ultimately DN.^31^, ^32^, ^33^ Research has indicated that DN patients with significant albuminuria exhibit reduced podocyte autophagy, and mice with podocyte-specific autophagy deficiency experience severe podocyte damage and excessive proteinuria in the presence of diabetes.^34^ These findings suggest that impaired autophagy in podocytes may contribute to podocyte dysfunction and the progression of DN. The protein junction p66Shc has been found to play an important role in regulating reactive oxygen species production and inducing apoptosis. Zheng et al^35^ found that the expression of p66Shc in podocytes of DN patients increased, while autophagy flux and the number of podocytes decreased. Animal and cellular experiments further demonstrated that p66Shc inhibits podocyte autophagy and induces apoptosis through the Notch-PTEN-PI3K/Akt/mTOR signaling pathway under high glucose conditions. Advanced glycation end products are the main factor of podocyte injury in DN. Zhao et al^36^ investigated the role and mechanism of advanced glycation end products in autophagy and found that their elevated levels led to excessive activation of mTOR, reduced nuclear translocation and activity of autophagy transcription factor EB, and decreased transcription of autophagy target genes, ultimately resulting in a decline in autophagy flux. However, overexpression of transcription factor EB could effectively prevent advanced glycation end product-induced autophagy. It is suggested that advanced glycation end products inhibit the formation and renewal of autophagosomes in podocytes by activating mTOR and inhibiting nuclear translocation of transcription factor EB, thus inducing podocyte injury. Liver X receptor is a metabolic nuclear receptor that is involved in various physiological processes. Its activation in podocytes has been shown to inhibit autophagy by disrupting the formation of autophagosomes *in vitro*. This effect may be mediated through the mTOR signaling pathway. In animal models, liver X receptor activation leads to inhibition of glomerular autophagy and worsens podocyte injury. It is suggested that its activation induces autophagy inhibition and leads to podocyte injury.^37^ In addition, the disorder of energy metabolism in a high glucose environment will affect the remodeling of the podocyte cytoskeleton and lead to podocyte dysfunction. Luo et al^38^ used metabonomic and transcriptome techniques to explore the metabolism of glucose, fat, and amino acids in cell and animal models and found that impaired podocyte glycolysis can enhance ornithine catabolism under diabetic conditions. Putrescine, one of the metabolites produced from ornithine catabolism, promotes mTOR signal activation and induces podocyte dysfunction by activating mTOR signal sensor GTPase Ras homologue in DN podocytes and remodeling the cytoskeleton. Other factors that have been implicated in inducing podocyte dysfunction in DN include up-regulation of complement factor B,^39^ increased levels of free fatty acids,^40^ and the overexpression of transforming growth factor β^41^ and Sestrin2,^42^ all of which activate the expression of mTOR and contribute to the development of DN. In summary, podocyte dysfunction plays a crucial role in the development and progression of DN. Impairment of podocyte autophagy, activation of p66shc, accumulation of advanced glycation end products, inhibition of autophagy by liver X receptor, disturbances in energy metabolism, and the involvement of various other factors all contribute to the damage and dysfunction of podocytes in DN (Fig. 5).

Podocytes serve as the ultimate barrier of the glomerular filtration barrier and additionally contribute significantly to the preservation of capillary support function, glomerular basement membrane repair, cellular biological function, and regulation of immune response.^43^ In the pathogenesis of DN, metabolic disturbances, inflammation, oxidative stress, and other factors can induce alterations in the structure and function of podocytes. Due to their terminally differentiated nature and limited regenerative capacity, podocytes are unable to undergo repair or replacement through cellular proliferation following injury, ultimately leading to irreversible glomerular damage. The presence of protein aggregates and organelle fragments in damaged podocytes can lead to the initiation of additional pathological mechanisms and expedite the progression of DN if not promptly eliminated. Consequently, the autophagy lysosomal system plays a vital role in upholding the stability of the podocyte's internal milieu.^44^ Previous research has demonstrated the molecular regulation of the mTOR signaling pathway and autophagy. Numerous investigations have been undertaken to investigate the interconnectedness between podocyte dysfunction and DN. Based on this premise, future investigations can delve into the alterations of additional small molecules within the mTOR signaling pathway, thereby elucidating the comprehensive mechanism underlying mTOR's implication in podocyte dysfunction.

### Glomerular mesangial cells and mTOR

Glomerular mesangial cells (GMCs) are cellular constituents found within the mesangial matrix of the interlobular axis of the glomerular capillary plexus. Their primary physiological roles encompass providing support to glomerular capillaries, regulating the surface area of the filtration membrane, and controlling the glomerular filtration coefficient. Additionally, these cells engage in phagocytosis of proteins and macromolecular metabolic wastes that become entrapped within the basement membrane of the glomerular filtration membrane, thereby contributing to the purification of the glomerular environment.^45^ Under the stimulation of high glucose, GMCs become abnormally activated, undergo abnormal proliferation, and deposit a large amount of extracellular matrix in the mesangial area, which eventually leads to glomerulosclerosis.^46^ This pathological change is closely related to the mTOR signal pathway and its mediated autophagy.^47^^,^^48^ Long-stranded non-coding RNA (lncRNA) has been found to play a significant role in the pathogenesis of human diseases, including DN. Previous research has demonstrated that lncRNA is involved in the development of DN and can regulate the proliferation, fibrosis, and inflammation of mesangial cells induced by high glucose levels.^49^^,^^50^ For instance, lncRNA HLA complex P5 (HCP5) is recognized as a carcinogenic driving factor in various cancers.^51^^,^^52^ In a study conducted by Wang et al,^53^ experiments were carried out to investigate the role of HCP5 in the progression of DN. Initially, bioinformatics analysis was utilized to identify miR-93-5p as a potential target of HCP5. Its role in renal interstitial fibrosis in DN has been reported.^54^ The results showed that in high glucose-induced human GMCs, HCP5 activates the AKT/mTOR pathway by regulating the miR-93-5p/HMGA2 axis, which leads to excessive proliferation, fibrosis, and inflammation of human GMCs. Moreover, Huang et al^55^ confirmed that lncRNA nuclear enriched abundant transcript 1 can promote mesangial cell proliferation, migration, and fibrosis by blocking PI3K/Akt/mTOR pathway. Transforming growth factor β also plays a regulatory role in the pathogenesis of various renal diseases, which can promote the occurrence and development of DN by inducing glomerulosclerosis and proteinuria.^56^^,^^57^ Maity et al^58^ found that transforming growth factor β can mediate miR-181a targeting inhibition of mTOR regulatory protein deptor, increase mTOR activity, and drive the proliferation and hypertrophy of mesangial cells. Furthermore, it can induce mTOR activation by phosphorylating platelet-derived growth factor receptor β, stimulating mesangial cell hypertrophy, and promoting matrix protein deposition.^59^ Additionally, the activation of mTOR can lead to increased production of reactive oxygen species by up-regulating antioxidant enzymes and down-regulating nicotinamide adenine dinucleotide phosphate oxidase activities. Consequently, this phenomenon can trigger apoptosis in GMCs and result in impairment of the glomerular filtration barrier^60^(Fig. 5).

In conclusion, the aberrant activation of mTOR led to the impairment of GMCs, thereby facilitating their proliferation and the secretion of mesangial matrix. This process ultimately led to the accumulation of collagen.^61^ Additionally, the compromised autophagy function contributed to the development of glomerular sclerosis and interstitial fibrosis, ultimately exacerbating the progression of DN.^62^ Effective inhibition of GMC dysfunction plays an important role in controlling the progression of DN. Previous studies have revealed that lncRNAs and microRNAs (miRs) participate in abnormal changes in the morphology and function of mesangial cells through interactions with the mTOR signaling pathway, as well as other factors such as transforming growth factor β and platelet-derived growth factor receptor β. A comprehensive comprehension of the correlation between mTOR and lncRNA, microRNA, and cytokines holds substantial importance in elucidating the exacerbated advancement of GMC injury in DN.

### Renal tubular epithelial cells and mTOR

Renal tubules, the primary structural units of the kidney, play a crucial role in various renal diseases, and their epithelial cells, known as renal tubular epithelial cells, are particularly important. Among the different regions of the renal tubules, the proximal tubule is the most active in terms of metabolic processes. It is responsible for recovering major nutrients, small proteins, electrolytes, and trace elements from the primary urine, which is essential for maintaining normal cell and tissue stability.^63^ Early indications of DN involve morphological changes in proximal tubular epithelial cells such as hypertrophy, proliferation, and epithelial–mesenchymal transition.^64^, ^65^, ^66^ Previous studies have revealed that the mTOR signaling pathway, a key regulatory pathway, can influence fibroblast proliferation, renal tubulointerstitial inflammation, and epithelial–mesenchymal transition under high glucose conditions.^67^^,^^68^ Tubulointerstitial fibrosis is a key feature of DN, and studies have shown that the expression of the key regulator of metabolic homeostasis called Yin Yang 1 (YY1) is significantly increased in proximal tubular epithelial cells under high glucose conditions. This increase leads to the formation of a protein complex called the mTOR-YY1 heterodimer, which induces mitochondrial dysfunction and oxidative stress, both of which are closely associated with tubulointerstitial fibrosis.^69^ Moreover, excessive production of reactive oxygen species due to oxidative stress can mediate epithelial–mesenchymal transition and inhibit the PI3K/Akt/mTOR pathway in renal tubular epithelial cells, promoting renal fibrosis.^70^ In recent years, miRs have emerged as important regulators that influence renal tubular cells and contribute to the development of DN. For example, miR-22 is shown to be up-regulated in renal tubular epithelial cells cultured with high glucose, leading to decreased expression of its target gene phosphatase and tensin homolog (PTEN). Abnormal miR-22 expression inhibits autophagy flux and induces the expression of collagen IV and alpha-smooth muscle actin, both of which contribute to fibrosis progression. However, the application of the mTOR inhibitor rapamycin, in combination with inhibiting miR-22 overexpression, can effectively alleviate autophagy inhibition and reduce the expression of collagen IV and alpha-smooth muscle actin, suggesting that miR-22 may cooperate with mTOR to promote tubulointerstitial fibrosis by targeting PTEN to partially inhibit autophagy.^71^ Furthermore, the abnormal expression of PTEN can promote the dysregulation of miR-214, leading to overexpression of mTOR and promoting hypertrophy and fibronectin expression in mesangial and proximal renal tubular epithelial cells under high glucose conditions.^72^ This study also demonstrated the positive feedback loop between the three-layer kinase cascade (PI3K/Akt/mTOR) and miR-214/PTEN, which contributes to the injury of renal tubular cells in a high glucose environment.^73^ Another study found that up-regulation of miR-214 significantly increases the expression of uncoupling protein 2, p-Akt, and p-mTOR in proximal tubular cells, leading to elevated levels of reactive oxygen species, oxidative stress, and cell injury.^74^ Additionally, miR-29c promotes DN renal fibrosis by activating the AMPK/mTOR signaling pathway, while overexpression of miR-136 down-regulates spleen tyrosine kinase and inhibits the transforming growth factor β1/Smad3 signaling pathway, leading to increased expression of mTOR and mediating renal tubular epithelial cell fibrosis.^75^^,^^76^ Moreover, serum and glucocorticoid-induced kinase 1 is known to up-regulate the phosphorylation of PI3K, AKT, and mTOR, inhibiting autophagy and promoting epithelial–mesenchymal transition in renal tubular epithelial cells.^77^ Another interesting finding is the significant correlation between a novel small molecule non-coding RNA called transfer RNA derived fragments and the accumulation of extracellular matrix in renal tubular epithelial cells induced by high glucose. Differentially expressed transfer RNA-derived fragments hold potential in unraveling the pathogenesis of DN (Fig. 5).^78^

Tubulointerstitial fibrosis generally exacerbates the initiation and advancement of DN, with epithelial–mesenchymal transition playing a crucial role. Following the epithelial–mesenchymal transition, renal tubular epithelial cells undergo a loss of their epithelial cell phenotype and acquire a mesenchymal cell phenotype, accompanied by a significant enhancement in their ability to synthesize and secrete extracellular matrix. Autophagy serves as a regulatory mechanism for epithelial–mesenchymal transition, safeguarding renal tubular epithelial cells.^79^ However, in a high-glucose environment, the inhibitory effects on autophagy function hinder its effectiveness in alleviating the progression of DN. mTOR plays an important role in the occurrence of this series of mechanisms. Previous studies have revealed many molecules involved in regulation. In the future, mTOR, tubulointerstitial fibrosis, and autophagy can be connected in tandem to conduct in-depth studies, further clarify the substances and molecules that play a role, and provide new ideas for the treatment of DN. mTOR assumes a crucial function in the manifestation of this sequence of mechanisms. Numerous molecules implicated in regulation have been unveiled by prior investigations. In subsequent research, the interconnection between mTOR, tubulointerstitial fibrosis, and autophagy can be explored in a sequential manner to undertake comprehensive studies, thereby elucidating the specific substances and molecules involved, and offering novel perspectives for the management of DN.

### Glomerular endothelial cells and mTOR

Glomerular endothelial cells serve as the first line of defense in the filtration process of the kidney. They form a continuous layer with small pores on their surface, allowing for the filtration of water molecules while selectively permitting the permeation of large macromolecular proteins. The surface of the glomerular endothelial cells is coated with a complex of negatively charged polysaccharides and proteins, which plays a critical role in maintaining the integrity of the glomerular filtration barrier.^80^ However, when these endothelial cells suffer damage, the negatively charged polysaccharide-protein complex is compromised, affecting its ability to effectively filter molecules. As a result, the defense mechanism weakens, and albuminuria, the presence of albumin in the urine, occurs. This further exacerbates renal dysfunction.^81^^,^^82^ One of the molecules implicated in causing endothelial cell damage is platelet microparticle-derived chemokine ligand 7.^83^ When present, it activates the mTOR signaling pathway, leading to the production of reactive oxygen species and a decrease in the level of nitric oxide. This, in turn, inhibits the activity of important enzymes like endothelial nitric oxide synthase and superoxide dismutase, ultimately increasing the permeability of the glomerular filtration barrier and promoting damage to the endothelial cells. In a study by Chen et al,^84^ it was discovered that under high glucose conditions, the activation of the mTOR signaling pathway up-regulates the expression and activity of a protein called RhoA. This leads to rearrangement of the endothelial cytoskeleton mediated by myosin light chain, resulting in dysfunction of the glomerular endothelial barrier. Other signaling pathways, such as the calcium/calmodulin-dependent protein kinase b/liver kinase B1/AMPK signal and the miR-34a/Atg4b signal, have also been identified as potential contributors to the progression of DN, providing additional targets for therapeutic interventions (Fig. 5).^85^^,^^86^

Glomerular endothelial cells play a crucial role in maintaining the integrity of the blood-tissue barrier and facilitating the transport of specific substances across membranes to support normal physiological functions. However, in the presence of high sugar levels, pathological conditions arise, characterized by the accumulation of inflammatory mediators and heightened permeability. These factors contribute to the disruption of the endothelial cell barrier, resulting in the formation of actin stress fibers and subsequent contraction of the endothelial cytoskeleton. Ultimately, these changes lead to an enlargement of the cell space.^87^ This process exacerbates the advancement of barrier dysfunction and DN. The aberrant activation of mTOR is intricately associated with the manifestation of these conditions, thus emphasizing the significance of conducting comprehensive investigations into the mTOR signaling pathway and uncovering additional factors that contribute to endothelial barrier dysfunction and cytoskeletal alignment.

### Immune cells and mTOR

Macrophages are mature cells in the mononuclear phagocytic system that are formed from monocytes in the blood and are essential for the immune response in tissues and organs.^88^ In the diabetic milieu, the kidney undergoes stimulation from elevated sugar levels, polyols, and advanced glycation end products, resulting in the secretion of substantial quantities of intercellular adhesion molecule-1, mononuclear/macrophage chemotactic protein-1, and fractalkine by endothelial cells.^89^ Research has shown that macrophages can migrate to the kidney through the actions of intercellular adhesion molecule-1, mononuclear/macrophage chemotactic protein-1, and fractalkine, where they infiltrate the renal tissue.^90^, ^91^, ^92^ The infiltration of macrophages is a characteristic of the inflammatory response in DN. Through the release of cytokines and inflammatory factors, macrophages can contribute to renal vascular endothelial cell injury, glomerular hypertrophy, and renal interstitial fibrosis, and are central to the development of DN.^93^ A study by Ren et al^94^ found that macrophage activation is closely linked to the mTOR pathway, and targeting this pathway in macrophages can alleviate renal fibrosis, suggesting it could be a potential avenue for preventing the progression of renal fibrosis in DN. T lymphocytes, also known as thymus-dependent lymphocytes, are found in the thymus-dependent areas of peripheral lymphoid tissues and are involved in both humoral and cellular immunity through circulation in the lymphatic vessels, peripheral blood, and interstitial fluid.^95^^,^^96^ Previous studies have observed T lymphocyte infiltration in the renal interstitium of both animal models and patients with DN.^97^ Similar to macrophages, the migration and infiltration of T lymphocytes to the kidney are influenced by intercellular adhesion molecule-1, mononuclear/macrophage chemotactic protein-1, and fractalkine. Activated T lymphocytes secrete inflammatory molecules such as interferon-γ and tumor necrosis factor β, which can activate endothelial cells and macroages, induce renal fibrosis, and contribute to the progression of DN.^98^ Furthermore, the up-regulation of DNA methyltransferase 1, a key enzyme involved in DNA methylation, in T lymphocytes can lead to abnormal methylation of upstream regulatory factors of the mTOR pathway, resulting in diabetic renal inflammation.^99^ The inhibitor of mTOR, rapamycin, has been found to significantly block the migration, infiltration, and activation of T lymphocytes, suggesting the interaction between the mTOR pathway and T lymphocytes plays a crucial role in the progression of DN.^100^ However, further research is needed to fully understand the specific mechanisms involved in this interaction (Fig. 5).

### Application strategy of mTOR in the treatment of DN

DN is a leading cause of chronic kidney disease and mortality among patients with diabetes. The development of DN begins with transient mesangial cell proliferation, which then progresses to cell hypertrophy and increased deposition of extracellular matrix. This ultimately leads to glomerulosclerosis, a characteristic feature of DN.^101^ Unfortunately, current treatment methods are not effective in significantly reducing the number of DN patients or slowing down the progression of the disease. Therefore, it is necessary to clarify the mechanism of diabetic renal damage and the changes in related signal pathways to develop new drugs. A number of studies have shown that the mTOR pathway is involved in the pathogenesis of DN. Activation of mTOR inhibits autophagy, promotes inflammation, induces oxidative stress, and contributes to the development of DN. Inhibition of the mTOR pathway may prevent the early structural changes in the kidney from progressing to full-blown DN. Numerous studies have demonstrated that rapamycin, a drug that targets mTOR, can inhibit the onset and progression of DN in animal models of diabetes.^102^, ^103^, ^104^ By inhibiting mTOR, rapamycin can alleviate podocyte damage, delay glomerular hypertrophy, and improve the pathological changes associated with DN. However, it should be noted that improper use of rapamycin can lead to several side effects, such as elevated urinary protein,^105^ acute renal tubular necrosis,^106^ insulin resistance, and abnormal lipid metabolism.^107^^,^^108^ Additionally, mTOR plays a crucial role in immune cell function, and the use of rapamycin may impact the immune system's ability to resist infections.^109^ Thiazolidinediones rosiglitazone and aldosterone antagonist spironolactone have also been found to delay the progression of early DN and improve the pathological changes associated with DN by modulating the mTOR pathway. Studies comparing rosiglitazone with rapamycin have shown that rosiglitazone is more effective in improving the renal pathological structure.^110^^,^^111^ Recent research has also shown that plant extracts and Chinese herbal medicines can interfere with the onset and progression of DN by regulating the mTOR pathway. For example, fucoidan, a natural marine polysaccharide, can inhibit podocyte pyrogenesis and reduce renal fibrosis in DN by regulating the AMPK/mTOR/NLRP3 signal axis.^112^ Emodin, a natural compound, can inhibit apoptosis, enhance podocyte autophagy, and alleviate renal fibrosis in DN rats by modulating the renal AMPK/mTOR signaling pathway.^113^ Arbutin, another natural compound, can regulate miR-27a to block the JNK and mTOR pathways and reduce apoptosis in renal cells treated with high glucose.^114^ Ginkgetin may reduce mesangial cell proliferation, oxidative stress, inflammation, and extracellular matrix accumulation induced by high glucose by activating the AMPK/mTOR-mediated autophagy pathway.^115^ Rutin has been shown to attenuate endothelial–interstitial transition by inhibiting the PI3K/AKT/mTOR pathway and restoring endothelial autophagy in DN.^116^ Tripterygium glycoside and triptolide can up-regulate podocyte autophagy through the mTOR pathway, thereby reducing epithelial–mesenchymal transition and apoptosis in DN.^117^^,^^118^ Other compounds, such as products of *Radix astragali*, *Paecilomyces Cicadidae*, dihydromyricetin, ginsenoside Rg1, and kaempferol, have also been found to protect the kidneys and delay the progression of DN by modulating mTOR-related pathways.^119^, ^120^, ^121^, ^122^ Furthermore, various molecules and proteins, including Salusin-α,^123^ Elabela,^124^ Klotho,^125^ and 1,25-dihydroxyvitamin D(3),^126^ have therapeutic effects that are closely linked to the mTOR pathway.

## Conclusion and outlook

In conclusion, the mTOR signaling pathway assumes a crucial role in the regulation of diverse pathophysiological processes associated with DN. Dysregulation of mTOR can exert an influence on the expression of other molecules, either independently or in conjunction with them, leading to the formation of complexes that subsequently modulate the mechanistic pathway, thereby inducing pathological alterations in four distinct types of kidney resident cells and immune cells. Consequently, this aberrant regulation contributes to the initiation and progression of DN. Furthermore, our investigation has revealed that the pathological mechanism associated with the mTOR signaling pathway exhibits variations across different cell types. Notably, autophagy inhibition is a shared effect observed in all four types of kidney cells. Autophagy is a biological phenomenon wherein cells undergo degradation of their internal organelles, proteins, and invading pathogens via lysosomes, as a response to external environmental stimuli and metabolic stressors. This process is crucial for the maintenance of cellular homeostasis and integrity, as it facilitates cell self-purification, remodeling, and the preservation of internal environmental equilibrium. Under normal physiological conditions, the majority of cells maintain a tightly regulated baseline level of autophagy to effectively eliminate and break down aberrant proteins and organelles, thereby ensuring cell survival and preserving the stability of the internal milieu. Nevertheless, in pathological scenarios, dysregulation of autophagy control disrupts the equilibrium, leading to the accumulation of abnormal proteins within cells or impaired organelle functionality. Consequently, this aberrant autophagy regulation ultimately gives rise to metabolic disturbances, oxidative stress, inflammation, and other pathological mechanisms, culminating in the impairment and demise of cellular function.^127^ In our study, we can see that autophagy is closely related to the occurrence and development of DN. In the physiological state, podocytes and tubular epithelial cells in the kidney are at a good level of autophagy and maintain the homeostasis of the renal environment. In the diabetic state, the presence of elevated glucose levels prompts the impairment of autophagy function, hindering the efficient clearance of damaged podocytes and renal tubular epithelial cells. Consequently, an accumulation of abnormal proteins and organelles transpires within the internal environment of the kidneys, leading to metabolic disturbances. Moreover, mesangial cells exposed to high glucose conditions exhibit structural and functional irregularities, thereby inducing abnormal secretion of the mesangial matrix. This, in turn, fosters the accumulation of collagen and fibronectin, exacerbating the onset of renal fibrosis. The disturbance of the internal environment and metabolic abnormalities stimulate endothelial cells, causing cytoskeletal rearrangement and affecting filtration barrier function. Simultaneously, endothelial cells secrete a large number of cytokines, inducing the migration of macrophages and T lymphocytes to the kidneys, resulting in immune infiltration and exacerbating inflammation and oxidative stress. It is through the collective action of these multiple mechanisms that the progression of DN is promoted. Hence, comprehending the intricate workings of the mTOR signaling pathway in its impact on the regulation of DN across various cell types holds immense importance for the advancement of efficacious therapeutic approaches in forthcoming endeavors. Future research should prioritize investigating the involvement of the mTOR signaling pathway and autophagy, as well as the intercellular cross-linking reaction, in order to elucidate the intricate pathogenesis of DN. We believe that through the continuous exploration of relevant studies, a more comprehensive elucidation of the interplay between the mTOR signaling pathway and kidney cells and immune cells will be achieved. This enhanced comprehension will yield valuable perspectives on the mechanisms underlying the progression of DN. Furthermore, armed with this understanding of the aforementioned interaction, novel approaches for the prevention and treatment of DN may be devised, potentially leading to improved prognoses for individuals afflicted by this grave diabetic complication. The continuation of research endeavors in this domain is anticipated to yield advancements in the management and prognostication of DN in the foreseeable future.

## Author contributions

Jingxuan Shi drafted the manuscript. Xinze Liu generated the figures. Yuanyuan Jiao and Jingwei Tian performed the background research. Jiaqi An and Guming Zou reviewed and edited the figures. Li Zhuo reviewed and edited the manuscript. All authors read and approved the content of the manuscript.

## Conflict of interests

The authors declare no conflict of interests.

## Funding

This study was supported by the Elite Medical Professionals Project of 10.13039/501100012173China-Japan Friendship Hospital (No. ZRJY2021-BJ07), the National High Level Hospital Clinical Research Funding (China) (No. 2023-NHLHCRF-PY-07), and the 10.13039/501100001809National Natural Science Foundation of China (No. 81870495).

## References

[1] Bell S., Fletcher E.H., Brady I. (2015). End-stage renal disease and survival in people with diabetes: a national database linkage study. QJM.

[2] Heerspink H.J.L., Parving H.H., Andress D.L. (2019). Atrasentan and renal events in patients with type 2 diabetes and chronic kidney disease (SONAR): a double-blind, randomised, placebo-controlled trial. Lancet.

[3] Ricciardi C.A., Gnudi L. (2021). Kidney disease in diabetes: from mechanisms to clinical presentation and treatment strategies. Metabolism.

[4] Alicic R.Z., Rooney M.T., Tuttle K.R. (2017). Diabetic kidney disease: challenges, progress, and possibilities. Clin J Am Soc Nephrol.

[5] Qi C., Mao X., Zhang Z., Wu H. (2017). Classification and differential diagnosis of diabetic nephropathy. J Diabetes Res.

[6] Yaribeygi H., Mohammadi M.T., Rezaee R., Sahebkar A. (2018). Crocin improves renal function by declining Nox-4, IL-18, and p53 expression levels in an experimental model of diabetic nephropathy. J Cell Biochem.

[7] Doshi S.M., Friedman A.N. (2017). Diagnosis and management of type 2 diabetic kidney disease. Clin J Am Soc Nephrol.

[8] Fried L.F., Emanuele N., Zhang J.H. (2013). Combined angiotensin inhibition for the treatment of diabetic nephropathy. N Engl J Med.

[9] Liu W.J., Huang W.F., Ye L. (2018). The activity and role of autophagy in the pathogenesis of diabetic nephropathy. Eur Rev Med Pharmacol Sci.

[10] Kitada M., Ogura Y., Monno I., Koya D. (2017). Regulating autophagy as a therapeutic target for diabetic nephropathy. Curr Diabetes Rep.

[11] Moreno J.A., Gomez-Guerrero C., Mas S. (2018). Targeting inflammation in diabetic nephropathy: a tale of hope. Expet Opin Invest Drugs.

[12] Vallon V., Komers R. (2011). Pathophysiology of the diabetic kidney. Compr Physiol.

[13] Grahammer F., Wanner N., Huber T.B. (2014). mTOR controls kidney epithelia in health and disease. Nephrol Dial Transplant.

[14] Lieberthal W., Levine J.S. (2012). Mammalian target of rapamycin and the kidney. II. Pathophysiology and therapeutic implications. Am J Physiol Ren Physiol.

[15] Inoki K., Mori H., Wang J. (2011). mTORC1 activation in podocytes is a critical step in the development of diabetic nephropathy in mice. J Clin Invest.

[16] Heitman J., Movva N.R., Hall M.N. (1991). Targets for cell cycle arrest by the immunosuppressant rapamycin in yeast. Science.

[17] Brown E.J., Albers M.W., Shin T.B. (1994). A mammalian protein targeted by G1-arresting rapamycin-receptor complex. Nature.

[18] Liu G.Y., Sabatini D.M. (2020). mTOR at the nexus of nutrition, growth, ageing and disease. Nat Rev Mol Cell Biol.

[19] Kajiwara M., Masuda S. (2016). Role of mTOR inhibitors in kidney disease. Int J Mol Sci.

[20] Wullschleger S., Loewith R., Hall M.N. (2006). TOR signaling in growth and metabolism. Cell.

[21] Jacinto E., Loewith R., Schmidt A. (2004). Mammalian TOR complex 2 controls the actin cytoskeleton and is rapamycin insensitive. Nat Cell Biol.

[22] Gödel M., Hartleben B., Herbach N. (2011). Role of mTOR in podocyte function and diabetic nephropathy in humans and mice. J Clin Invest.

[23] Puelles V.G., van der Wolde J.W., Wanner N. (2019). mTOR-mediated podocyte hypertrophy regulates glomerular integrity in mice and humans. JCI Insight.

[24] Qiao S., Liu R., Lv C. (2019). Bergenin impedes the generation of extracellular matrix in glomerular mesangial cells and ameliorates diabetic nephropathy in mice by inhibiting oxidative stress via the mTOR/β-TrcP/Nrf2 pathway. Free Radic Biol Med.

[25] Mohan T., Narasimhan K.K.S., Ravi D.B. (2020). Role of Nrf2 dysfunction in the pathogenesis of diabetic nephropathy: therapeutic prospect of epigallocatechin-3-gallate. Free Radic Biol Med.

[26] Bao L., Li J., Zha D. (2018). Chlorogenic acid prevents diabetic nephropathy by inhibiting oxidative stress and inflammation through modulation of the Nrf2/HO-1 and NF-κB pathways. Int Immunopharm.

[27] Nagata M. (2016). Podocyte injury and its consequences. Kidney Int.

[28] Hartleben B., Gödel M., Meyer-Schwesinger C. (2010). Autophagy influences glomerular disease susceptibility and maintains podocyte homeostasis in aging mice. J Clin Invest.

[29] Hong Q., Zhang L., Das B. (2018). Increased podocyte Sirtuin-1 function attenuates diabetic kidney injury. Kidney Int.

[30] Ding Y., Choi M.E. (2015). Autophagy in diabetic nephropathy. J Endocrinol.

[31] Wang W., Sun W., Cheng Y., Xu Z., Cai L. (2019). Role of sirtuin-1 in diabetic nephropathy. J Mol Med.

[32] Lin T.A., Wu V.C.C., Wang C.Y. (2019). Autophagy in chronic kidney diseases. Cells.

[33] Liu F., Guo J., Qiao Y. (2021). miR-138 plays an important role in diabetic nephropathy through SIRT1-p38-TTP regulatory axis. J Cell Physiol.

[34] Tagawa A., Yasuda M., Kume S. (2016). Impaired podocyte autophagy exacerbates proteinuria in diabetic nephropathy. Diabetes.

[35] Zheng D., Tao M., Liang X., Li Y., Jin J., He Q. (2020). p66Shc regulates podocyte autophagy in high glucose environment through the Notch-PTEN-PI3K/Akt/mTOR pathway. Histol Histopathol.

[36] Zhao X., Chen Y., Tan X. (2018). Advanced glycation end-products suppress autophagic flux in podocytes by activating mammalian target of rapamycin and inhibiting nuclear translocation of transcription factor EB. J Pathol.

[37] Zhang Z., Tang S., Gui W. (2020). Liver X receptor activation induces podocyte injury via inhibiting autophagic activity. J Physiol Biochem.

[38] Luo Q., Liang W., Zhang Z. (2022). Compromised glycolysis contributes to foot process fusion of podocytes in diabetic kidney disease: role of ornithine catabolism. Metabolism.

[39] Lu Q., Hou Q., Cao K. (2021). Complement factor B in high glucose-induced podocyte injury and diabetic kidney disease. JCI Insight.

[40] Denhez B., Rousseau M., Spino C. (2020). Saturated fatty acids induce insulin resistance in podocytes through inhibition of IRS1 via activation of both IKKβ and mTORC1. Sci Rep.

[41] Das F., Ghosh-Choudhury N., Lee D.Y., Gorin Y., Kasinath B.S., Choudhury G.G. (2018). Akt2 causes TGFβ-induced deptor downregulation facilitating mTOR to drive podocyte hypertrophy and matrix protein expression. PLoS One.

[42] Ala M. (2023). Sestrin2 signaling pathway regulates podocyte biology and protects against diabetic nephropathy. J Diabetes Res.

[43] Ichimura K., Miyaki T., Kawasaki Y., Kinoshita M., Kakuta S., Sakai T. (2019). Morphological processes of foot process effacement in puromycin aminonucleoside nephrosis revealed by FIB/SEM tomography. J Am Soc Nephrol.

[44] Fang L., Zhou Y., Cao H. (2013). Autophagy attenuates diabetic glomerular damage through protection of hyperglycemia-induced podocyte injury. PLoS One.

[45] Lu X., Zhu X., Yu M., Na C., Gan W., Zhang A. (2019). Profile analysis reveals transfer RNA fragments involved in mesangial cells proliferation. Biochem Biophys Res Commun.

[46] Wu Z., Yin W., Sun M., Si Y., Wu X., Chen M. (2020). BK_Ca_ mediates dysfunction in high glucose induced mesangial cell injury via TGF-*β*1/Smad2/3 signaling pathways. Internet J Endocrinol.

[47] Wang X., Gao Y., Tian N. (2018). Astragaloside IV represses high glucose-induced mesangial cells activation by enhancing autophagy via SIRT1 deacetylation of NF-κB p65 subunit. Drug Des Dev Ther.

[48] Gao C., Fan F., Chen J. (2019). FBW_7_ regulates the autophagy signal in mesangial cells induced by high glucose. BioMed Res Int.

[49] Wang S., Chen X., Wang M. (2018). Long non-coding RNA CYP4B1-PS1-001 inhibits proliferation and fibrosis in diabetic nephropathy by interacting with nucleolin. Cell Physiol Biochem.

[50] Zhang P., Sun Y., Peng R. (2019). Long non-coding RNA Rpph1 promotes inflammation and proliferation of mesangial cells in diabetic nephropathy via an interaction with Gal-3. Cell Death Dis.

[51] Jiang L., Wang R., Fang L. (2019). HCP_5_ is a SMAD3-responsive long non-coding RNA that promotes lung adenocarcinoma metastasis via miR-203/SNAI axis. Theranostics.

[52] Liang L., Xu J., Wang M. (2018). LncRNA HCP_5_ promotes follicular thyroid carcinoma progression via miRNAs sponge. Cell Death Dis.

[53] Wang X., Liu Y., Rong J., Wang K. (2021). LncRNA HCP_5_ knockdown inhibits high glucose-induced excessive proliferation, fibrosis and inflammation of human glomerular mesangial cells by regulating the miR-93-5p/HMGA2 axis. BMC Endocr Disord.

[54] Yang J., Shen Y., Yang X. (2019). Silencing of long noncoding RNA XIST protects against renal interstitial fibrosis in diabetic nephropathy via microRNA-93-5p-mediated inhibition of CDKN1A. Am J Physiol Ren Physiol.

[55] Huang S., Xu Y., Ge X. (2019). Long noncoding RNA NEAT1 accelerates the proliferation and fibrosis in diabetic nephropathy through activating Akt/mTOR signaling pathway. J Cell Physiol.

[56] Kanwar Y.S., Sun L., Xie P., Liu F.Y., Chen S. (2011). A glimpse of various pathogenetic mechanisms of diabetic nephropathy. Annu Rev Pathol.

[57] Kanwar Y.S., Jun W., Lin S. (2008). Diabetic nephropathy: mechanisms of renal disease progression. Exp Biol Med.

[58] Maity S., Bera A., Ghosh-Choudhury N., Das F., Kasinath B.S., Choudhury G.G. (2018). microRNA-181a downregulates deptor for TGFβ-induced glomerular mesangial cell hypertrophy and matrix protein expression. Exp Cell Res.

[59] Maity S., Das F., Kasinath B.S., Ghosh-Choudhury N., Ghosh Choudhury G. (2020). TGFβ acts through PDGFRβ to activate mTORC1 via the Akt/PRAS40 axis and causes glomerular mesangial cell hypertrophy and matrix protein expression. J Biol Chem.

[60] Lu Q., Zhou Y., Hao M. (2018). The mTOR promotes oxidative stress-induced apoptosis of mesangial cells in diabetic nephropathy. Mol Cell Endocrinol.

[61] Xiang H., Xue W., Wu X. (2019). FOXP1 inhibits high glucose-induced ECM accumulation and oxidative stress in mesangial cells. Chem Biol Interact.

[62] Hartleben B., Wanner N., Huber T.B. (2014). Autophagy in glomerular health and disease. Semin Nephrol.

[63] Klootwijk E.D., Reichold M., Unwin R.J., Kleta R., Warth R., Bockenhauer D. (2015). Renal Fanconi syndrome: taking a proximal look at the nephron. Nephrol Dial Transplant.

[64] Lee Y.H., Kim S.H., Kang J.M. (2019). Empagliflozin attenuates diabetic tubulopathy by improving mitochondrial fragmentation and autophagy. Am J Physiol Ren Physiol.

[65] Haraguchi R., Kohara Y., Matsubayashi K., Kitazawa R., Kitazawa S. (2020). New insights into the pathogenesis of diabetic nephropathy: proximal renal tubules are primary target of oxidative stress in diabetic kidney. Acta Histochem Cytoc.

[66] Wu M., Zhang M., Zhang Y. (2021). Relationship between lysosomal dyshomeostasis and progression of diabetic kidney disease. Cell Death Dis.

[67] Lieberthal W., Levine J.S. (2009). The role of the mammalian target of rapamycin (mTOR) in renal disease. J Am Soc Nephrol.

[68] Kume S. (2019). Pathophysiological roles of nutrient-sensing mechanisms in diabetes and its complications. Diabetol Int.

[69] Yang T., Hu Y., Chen S. (2023). YY1 inactivated transcription co-regulator PGC-1α to promote mitochondrial dysfunction of early diabetic nephropathy-associated tubulointerstitial fibrosis. Cell Biol Toxicol.

[70] Lu Q., Wang W.W., Zhang M.Z. (2019). ROS induces epithelial-mesenchymal transition via the TGF-β1/PI3K/Akt/mTOR pathway in diabetic nephropathy. Exp Ther Med.

[71] Zhang Y., Zhao S., Wu D. (2018). MicroRNA-22 promotes renal tubulointerstitial fibrosis by targeting PTEN and suppressing autophagy in diabetic nephropathy. J Diabetes Res.

[72] Bera A., Das F., Ghosh-Choudhury N., Mariappan M.M., Kasinath B.S., Ghosh Choudhury G. (2017). Reciprocal regulation of miR-214 and PTEN by high glucose regulates renal glomerular mesangial and proximal tubular epithelial cell hypertrophy and matrix expansion. Am J Physiol Cell Physiol.

[73] Maity S., Das F., Ghosh-Choudhury N., Kasinath B.S., Ghosh Choudhury G. (2019). High glucose increases miR-214 to power a feedback loop involving PTEN and the Akt/mTORC1 signaling axis. FEBS Lett.

[74] Yang S., Fei X., Lu Y., Xu B., Ma Y., Wan H. (2019). miRNA-214 suppresses oxidative stress in diabetic nephropathy via the ROS/Akt/mTOR signaling pathway and uncoupling protein 2. Exp Ther Med.

[75] Shao H., Huang Y., Hu H.L., Fan W.X., Yin X.N. (2019). Effect of miR-29c on renal fibrosis in diabetic rats via the AMPK/mTOR signaling pathway. Eur Rev Med Pharmacol Sci.

[76] Liu L., Pang X., Shang W., Feng G., Wang Z., Wang J. (2020). miR-136 improves renal fibrosis in diabetic rats by targeting down-regulation of tyrosine kinase SYK and inhibition of TGF-β1/Smad3 signaling pathway. Ren Fail.

[77] Zhuang L., Jin G., Hu X., Yang Q., Shi Z. (2019). The inhibition of SGK1 suppresses epithelial-mesenchymal transition and promotes renal tubular epithelial cell autophagy in diabetic nephropathy. Am J Transl Res.

[78] Ji J., Rong J., Zheng H. (2022). Expression profiles of tRNA-derived fragments in high glucose-treated tubular epithelial cells. Exp Ther Med.

[79] Xie Z., Chen Z., Chen J. (2021). Combination therapy with Exendin-4 and islet transplantation as a synergistic treatment for diabetic nephropathy in rats. Life Sci.

[80] Jeansson M., Haraldsson B. (2006). Morphological and functional evidence for an important role of the endothelial cell glycocalyx in the glomerular barrier. Am J Physiol Ren Physiol.

[81] Matsuda J., Namba T., Takabatake Y. (2018). Antioxidant role of autophagy in maintaining the integrity of glomerular capillaries. Autophagy.

[82] Gui Z., Suo C., Wang Z. (2021). Impaired ATG16L-dependent autophagy promotes renal interstitial fibrosis in chronic renal graft dysfunction through inducing EndMT by NF-κB signal pathway. Front Immunol.

[83] Zhang Y., Ma K.L., Gong Y.X. (2018). Platelet microparticles mediate glomerular endothelial injury in early diabetic nephropathy. J Am Soc Nephrol.

[84] Chen X., Chen J., Li X., Yu Z. (2021). Activation of mTOR mediates hyperglycemia-induced renal glomerular endothelial hyperpermeability via the RhoA/ROCK/pMLC signaling pathway. Diabetol Metab Syndrome.

[85] Lim J.H., Kim H.W., Kim M.Y. (2018). Cinacalcet-mediated activation of the CaMKKβ-LKB1-AMPK pathway attenuates diabetic nephropathy in db/db mice by modulation of apoptosis and autophagy. Cell Death Dis.

[86] Hao J., Liu X., Tang J. (2022). The effect of allograft inflammatory factor-1 on inflammation, oxidative stress, and autophagy via miR-34a/ATG4B pathway in diabetic kidney disease. Oxid Med Cell Longev.

[87] Radeva M.Y., Waschke J. (2018). Mind the gap: mechanisms regulating the endothelial barrier. Acta Physiol.

[88] Zeng L.F., Xiao Y., Sun L. (2019). A glimpse of the mechanisms related to renal fibrosis in diabetic nephropathy. Adv Exp Med Biol.

[89] Engel J.E., Chade A.R. (2019). Macrophage polarization in chronic kidney disease: a balancing act between renal recovery and decline?. Am J Physiol Ren Physiol.

[90] Calle P., Hotter G. (2020). Macrophage phenotype and fibrosis in diabetic nephropathy. Int J Mol Sci.

[91] Yang Z., Guo Z., Dong J. (2018). miR-374a regulates inflammatory response in diabetic nephropathy by targeting MCP-1 expression. Front Pharmacol.

[92] Navarro-González J.F., Mora-Fernández C., Muros de Fuentes M., García-Pérez J. (2011). Inflammatory molecules and pathways in the pathogenesis of diabetic nephropathy. Nat Rev Nephrol.

[93] Tesch G.H. (2017). Diabetic nephropathy - is this an immune disorder?. Clin Sci.

[94] Ren J., Li J., Feng Y. (2017). Rictor/mammalian target of rapamycin complex 2 promotes macrophage activation and kidney fibrosis. J Pathol.

[95] Bending J.J., Lobo-Yeo A., Vergani D., Viberti G.C. (1988). Proteinuria and activated T-lymphocytes in diabetic nephropathy. Diabetes.

[96] Bruserud O., Pawelec G. (1997). Interleukin-13 secretion by normal and posttransplant T lymphocytes; *in vitro* studies of cellular immune responses in the presence of acute leukaemia blast cells. Cancer Immunol Immunother.

[97] Wu C.C., Sytwu H.K., Lu K.C., Lin Y.F. (2011). Role of T cells in type 2 diabetic nephropathy. Exp Diabetes Res.

[98] Gao Q., Shen W., Qin W. (2010). Treatment of db/db diabetic mice with triptolide: a novel therapy for diabetic nephropathy. Nephrol Dial Transplant.

[99] Chen G., Chen H., Ren S. (2019). Aberrant DNA methylation of mTOR pathway genes promotes inflammatory activation of immune cells in diabetic kidney disease. Kidney Int.

[100] Powell J.D., Pollizzi K.N., Heikamp E.B., Horton M.R. (2012). Regulation of immune responses by mTOR. Annu Rev Immunol.

[101] Young B.A., Johnson R.J., Alpers C.E. (1995). Cellular events in the evolution of experimental diabetic nephropathy. Kidney Int.

[102] Kogot-Levin A., Hinden L., Riahi Y. (2020). Proximal tubule mTORC1 is a central player in the pathophysiology of diabetic nephropathy and its correction by SGLT2 inhibitors. Cell Rep.

[103] Reifsnyder P.C., Flurkey K., Te A., Harrison D.E. (2016). Rapamycin treatment benefits glucose metabolism in mouse models of type 2 diabetes. Aging.

[104] Mori H., Inoki K., Masutani K. (2009). The mTOR pathway is highly activated in diabetic nephropathy and rapamycin has a strong therapeutic potential. Biochem Biophys Res Commun.

[105] Murakami N., Riella L.V., Funakoshi T. (2014). Risk of metabolic complications in kidney transplantation after conversion to mTOR inhibitor: a systematic review and meta-analysis. Am J Transplant.

[106] Paluri R.K., Sonpavde G., Morgan C., Rojymon J., Mar A.H., Gangaraju R. (2019). Renal toxicity with mammalian target of rapamycin inhibitors: a meta-analysis of randomized clinical trials. Oncol Rev.

[107] Laplante M., Sabatini D.M. (2012). mTOR signaling in growth control and disease. Cell.

[108] Lamming D.W., Sabatini D.M. (2013). A central role for mTOR in lipid homeostasis. Cell Metabol.

[109] Shi G., Ozog S., Torbett B.E., Compton A.A. (2018). mTOR inhibitors lower an intrinsic barrier to virus infection mediated by IFITM3. Proc Natl Acad Sci U S A.

[110] Flaquer M., Lloberas N., Franquesa M. (2010). The combination of sirolimus and rosiglitazone produces a renoprotective effect on diabetic kidney disease in rats. Life Sci.

[111] Li D., Lu Z., Xu Z. (2016). Spironolactone promotes autophagy via inhibiting PI3K/AKT/mTOR signalling pathway and reduce adhesive capacity damage in podocytes under mechanical stress. Biosci Rep.

[112] Wang M.Z., Wang J., Cao D.W. (2022). Fucoidan alleviates renal fibrosis in diabetic kidney disease *via* inhibition of NLRP3 inflammasome-mediated podocyte pyroptosis. Front Pharmacol.

[113] Liu H., Wang Q., Shi G. (2021). Emodin ameliorates renal damage and podocyte injury in a rat model of diabetic nephropathy via regulating AMPK/mTOR-mediated autophagy signaling pathway. Diabetes Metab Syndr Obes.

[114] Lv L., Zhang J., Tian F., Li X., Li D., Yu X. (2019). Arbutin protects HK-2 cells against high glucose-induced apoptosis and autophagy by up-regulating microRNA-27a. Artif Cells, Nanomed Biotechnol.

[115] Lin W., Pan J., Huang E., Zhu Q. (2021). Ginkgetin alleviates high glucose-evoked mesangial cell oxidative stress injury, inflammation, and extracellular matrix (ECM) deposition in an AMPK/mTOR-mediated autophagy axis. Chem Biol Drug Des.

[116] Dong R., Zhang X., Liu Y. (2023). Rutin alleviates EndMT by restoring autophagy through inhibiting HDAC1 via PI3K/AKT/mTOR pathway in diabetic kidney disease. Phytomedicine.

[117] Tao M., Zheng D., Liang X. (2021). *Tripterygium* glycoside suppresses epithelial-to-mesenchymal transition of diabetic kidney disease podocytes by targeting autophagy through the mTOR/Twist1 pathway. Mol Med Rep.

[118] Li X.Y., Wang S.S., Han Z. (2017). Triptolide restores autophagy to alleviate diabetic renal fibrosis through the miR-141-3p/PTEN/akt/mTOR pathway. Mol Ther Nucleic Acids.

[119] Yang F., Qu Q., Zhao C. (2020). *Paecilomyces cicadae*-fermented *Radix astragali* activates podocyte autophagy by attenuating PI3K/AKT/mTOR pathways to protect against diabetic nephropathy in mice. Biomed Pharmacother.

[120] Guo L., Tan K., Luo Q., Bai X. (2020). Dihydromyricetin promotes autophagy and attenuates renal interstitial fibrosis by regulating miR-155-5p/PTEN signaling in diabetic nephropathy. Bosn J Basic Med Sci.

[121] Wang T., Gao Y., Yue R. (2020). Ginsenoside Rg1 alleviates podocyte injury induced by hyperlipidemia via targeting the mTOR/NF-*κ*B/NLRP3 axis. Evid Based Complement Alternat Med.

[122] Sheng H., Zhang D., Zhang J. (2022). Kaempferol attenuated diabetic nephropathy by reducing apoptosis and promoting autophagy through AMPK/mTOR pathways. Front Med.

[123] Wang W.J., Jiang X., Gao C.C., Chen Z.W. (2022). Salusin-α mitigates diabetic nephropathy via inhibition of the Akt/mTORC1/p70S6K signaling pathway in diabetic rats. Drug Chem Toxicol.

[124] Zhang Y., Wang Y., Luo M. (2019). Elabela protects against podocyte injury in mice with streptozocin-induced diabetes by associating with the PI3K/Akt/mTOR pathway. Peptides.

[125] Wu C., Ma X., Zhou Y., Liu Y., Shao Y., Wang Q. (2019). Klotho restraining Egr1/TLR4/mTOR axis to reducing the expression of fibrosis and inflammatory cytokines in high glucose cultured rat mesangial cells. Exp Clin Endocrinol Diabetes.

[126] Chen D.P., Ma Y.P., Zhuo L. (2017). 1, 25-Dihydroxyvitamin D_3_ inhibits the proliferation of rat mesangial cells induced by high glucose via DDIT4. Oncotarget.

[127] Klionsky D.J., Abdel-Aziz A.K., Abdelfatah S. (2021). Guidelines for the use and interpretation of assays for monitoring autophagy (4th edition). Autophagy.

